# Molecular basis of vascular events following spinal cord injury

**Published:** 2010-08-25

**Authors:** C Sinescu, F Popa, VT Grigorean, G Onose, A Sandu, M Popescu, G Burnei, V Strambu, C Popa

**Affiliations:** * Carol Davila University of Medicine and Pharmacy, BucharestRomania; ** ‘Bagdasar–Arseni’ Clinical Emergency Hospital, Department of Cardiology, BucharestRomania; *** ‘St. Pantelimon’ Clinical Emergency Hospital, Department of General Surgery, BucharestRomania; **** ‘Bagdasar–Arseni’ Clinical Emergency Hospital, Department of General Surgery, BucharestRomania; **** ‘Bagdasar–Arseni’ Clinical Emergency Hospital, Department of Physical and Rehabilitation Medicine, BucharestRomania; ***** ‘Bagdasar–Arseni’ Clinical Emergency Hospital, Department of Neurosurgery, BucharestRomania; ******* District Hospital Pitesti, Department of Neurosurgery, PitestiRomania; ******** ‘Marie Sklodowska Curie’ Children' Clinical Emergency Hospital, Department of Pediatric Orthopedic Surgery, BucharestRomania; ********* National Institute of Neurology and Neurovascular Disorders, Department of Neurology, BucharestRomania

**Keywords:** intraparenchymal hemorrhage, heme oxygenase 1, heme oxygenase 2, posttraumatic inflammatory response, blood–spinal cord barrier, endothelin 1, matrix metalloproteinasis

## Abstract

The aim of this article is to analyze the effects of the molecular basis of vascular events following spinal cord injury and their contribution in pathogenesis.

First of all, we reviewed the anatomy of spinal cord vessels.

The pathophysiology of spinal cord injuries revealed two types of pathogenic mechanisms. The primary event, the mechanic trauma, results in a disruption of neural and vascular structures into the spinal cord. It is followed by secondary pathogenesis that leads to the progression of the initial lesion. We reviewed vascular responses following spinal cord injury, focusing on both primary and secondary events. The intraparenchymal hemorrhage is a direct consequence of trauma; it has a typical pattern of distribution into the contused spinal cord, inside the gray matter and, it is radially extended into the white matter. The intraparenchymal hemorrhage is restricted to the dorsal columns, into adjacent rostral and caudal spinal segments. Distribution of chronic lesions overlaps the pattern of the early intraparenchymal hemorrhage. We described the mechanisms of action, role, induction and distribution of the heme oxygenase isoenzymes 1 and 2. Posttraumatic inflammatory response contributes to secondary pathogenesis. We analyzed the types of cells participating in the inflammatory response, the moment of appearance after the injury, the decrease in number, and the nature of their actions. The disruption of the blood–spinal cord barrier is biphasic. It exposes the spinal cord to inflammatory cells and to toxic effects of other molecules. Endothelin 1 mediates oxidative stress into the spinal cord through the modulation of spinal cord blood flow. The role of matrix metalloproteinases in blood–spinal cord barrier disruption, inflammation, and angiogenesis are reviewed.

The spinal cord injury is one of most important health problems worldwide, and one of the most devastating of all traumatic events, with an annual incidence of 15 to 50 cases per million of population. About 80% are young males, aged between 15 and 35 years, and 5% are children.[1–4] 

The aim of this article is the evaluation of the role of molecular basis of vascular disturbances in the pathophysiology of spinal cord injury. Clinical and experimental studies showed the existence of two types of mechanisms, primary and secondary, in spinal cord injury pathophysiology. Primary pathophysiological mechanism and mechanical trauma result in direct disruption of neural and vascular structures. The initial trauma is followed by secondary pathogenic mechanisms, including various biochemical and molecular events, which contribute to the progression of the primary traumatic lesion. Vascular disturbances pay a very important role in both primary and secondary pathophysiological events following spinal cord injury.[5] 

## Spinal cord vascular system

### Arterial supply

The arterial blood supply of the spinal cord is provided by anterior and posterior spinal arteries, descending branches from vertebral arteries, and by anterior and posterior radicular arteries, arising from segmental vessels.  

Anterior spinal artery, has a descending trajectory, and is located in a pial twofold, along the anterior median fissure. It supplies the anterior two thirds of the cord. Posterior spinal arteries are located behind the dorsal root of the spinal nerve. They supply the posterior one third of the cord.  

Radicular arteries are branches from segmental vessels (ascending cervical, deep cervical, intercostal, lumbar, and sacral arteries), that enter the vertebral canal, passing through intervertebral foramina and give rise to anterior and posterior radicular branches and a menigeal branch. Segmental radicular arteries supply roots and cord. Artery of Adamkiewicz is the main source of irrigation from T8 to conus medullaris. It usually originates between T9 and L2 and it is found in 85% of cases, and between T5 and T8 in 15% of cases. It supplies the anterior two thirds of the lower two thirds of the cord. 

The anastomoses among the anterior and posterior radicular arteries and anterior and posterior spinal arteries form an anastomotic pial plexus vasocorona.

Central arteries, originating from anterior spinal artery, posterior spinal arteries and vasocorona, represent intrinsic arteries.  The greatest density of central arteries is found in the cervical region, 8–13 arteries per centimeter, and there are only 2–3 arteries per centimeter in the thoracolumbar region. 

Mainly, there are two vascular territories: the anterior two thirds of the cord supplied by anterior spinal artery, central arteries and vasocorona, and the posterior one third supplied by posterior spinal arteries and vasocorona. Spinal cord blood flow is not entirely unidirectional. In spinal injury, multidirectional flow protects the cord from ischemia, by inverting the flow and diverting blood to affected areas.  

Watershed zones are the most vulnerable areas in the case of spinal cord injury. In the thoracic region, the vessels have a smaller caliber and there are a few anastomoses. Ischemic disturbances, which lead to aggravating neurological deficits, are frequently noted in superior thoracic (T1–T4) and thoracolumbar (T12–L1) spinal cord injury. Another watershed zone is described between the centrifugal system, derived from the central artery and centripetal system, formed by vasocorona.  

### Venous drainage

A venous net is formed at the pial level; it collects the blood into six venous channels located along the anterior median fissure, posterior median sulcus and behind the roots of the spinal nerves. 

## Intraparenchymal hemorrhage

The immediate posttraumatic effect in the spinal cord injury is the vasospasm of the superficial vessels, leading to intraparenchymal hemorrhage, with propensity for highly vascularized areas and for central gray matter.[6,7] The mechanical trauma causes disruption of gray matter microvasculature, diminishes perfusion, and impairs autoregulation of the spinal cord blood flow. The diminishing of the spinal cord blood supply is maintained or aggravated by systemic responses including: neurogenic shock, arterial hypotension, bradycardia, aritmia or hemorrhagic shock. 

The size of the intraparenchymal hemorrhage in the acute phase is directly proportional with the severity of the initial impact. In the acute contused cord, in 3–5 days time after the injury, the intraparenchymal hemorrhage is maximal to the injury site, and extends into the rostral and caudal spinal segments. Intraparenchymal hemorrhage is found centrally to the injury site, into the gray matter and in the adjacent white matter, radially oriented. At distant sites, intraparechymal hemorrhage occupies the central part of the dorsal column.[5,7] Necrosis is the consequence of intraparenchymal hemorrhage. Blood flow diminishes adjacent to hemorrhagic areas, resulting in different grades of ischemia. Some other events causing ischemia are represented by vasogenic edema secondary to blood–spinal cord barrier breakdown, direct compression of the adjacent structures by mass effect, direct vasospasm following mechanical trauma, or secondary vasospasm due to the exposure to heme degradation products and endothelin 1. The white matter surrounding the hemorrhagic gray matter shows a variety of degenerative lesions, including disrupted myelin, axonal and periaxonal swelling. [5] 

In the chronic phase, in 3–9 months time after the trauma, there are large, fusiform–shaped, well–demarcated cavitations into the injured cord, with maximum size at the epicenter of the lesion, with central myelomalacia appearance and extending into the dorsal columns in rostral and caudal segments. The same pattern of injury is maintained during the chronic stage, intramedullary cavities corresponding to regions exhibiting acute posttraumatic intraparenchymal hemorrhage. [5,7–9] The maintenance of the injury pattern proves that chronic lesions are the results of intraparenchymal hemorrhage.10 Extensive loss of white matter is a result of the obstruction of intramedullary vessels, due to initial trauma or secondary pathogenic events.[5,7] 

Reperfusion of ischemic tissue or hypoperfusion generates free radicals. Phagocytic cells activation and metal ions releasing, during the hem degradation leads to free radicals formation. Central nervous system is highly sensitive to free radicals effects. Cellular membranes made of polysaturated fatty acid chains are sensitive to free radicals actions. Besides, the central nervous system has limited defense mechanisms. Catalase, superoxide dismutase, and glutathione peroxidase activity is reduced in the central nervous system.[11] 

### Heme oxygenase 

Experimental studies showed neuronal and glial response to the posttraumatic intraparenchymal hemorrhage. Heme oxygenase is an enzyme, which degrades hem into equimolar quantities of biliverdin, carbon monoxide, and free iron (Fe^3+^).[12,13] Serin–treonin kinase and biliverdin–reductase catalyzes biliverdin reduction into bilirubin. This pathway is particularly important because bile pigments have antioxidant effects and protect cells against free radical-mediated damage. 

Three heme oxygenase isoenzymes have been described: heme oxygenase 1, heme oxygenase 2 and heme oxygenase 3.

Heme oxygenase 1, the inducible form of heme oxygenase, is an important defense mechanism against early oxidative stress, by stabilizing the blood–spinal cord barrier and limiting the infiltration of leukocytes.[14] In normal tissue, heme oxygenase 1 is found exclusively in neurons.[15,16] Posttraumatic,  heme oxigenase 1 is induced in gray matter as well as in white matter to the injury site. Heme oxygenase 1 is induced in the dorsal columns and, occasionally, in the white matter in segments rostral and caudal to the contused cord, on a length of 1 cm.[15,17] The distribution of cells containing heme oxygenase match the pattern of intraparenchymal hemorrhage. This proves acute induction of heme oxygenase in other cells too, besides neurons. [15,17] The acute induction of heme oxygenase 1 in spinal cord injury is found also in microglia, astrocytes and macrophages.[15] The induction of heme oxigenase 1 in glial cells may be a consequence of multiple factors. Bleeding is the main cause of heme oxygenase induction, by exposure at hem and hem products of degradation. Hypoxia, oxidative stress, and endothelin 1 exposure also lead to induction of the enzyme.[7,15,17,18] Consequences of heme oxygenase 1 induction in spinal cord injury are still on debate. It seems that the induction of this enzyme might have a protective role on contused spinal cord.[7] 

Heme oxygenase 2 is a constitutive form, which maintains intracellular hem homeostasis  and inactivates nitrosyl–derived free radicals.[7,16,19] Heme oxygenase 2 binds free radicals at heme regulatory motifs, and it is inactivated afterwards at protein and transcript levels. Heme oxygenase 2 was found in ventral horn motor neurons, oligodendroglia, and ependymal cells.[20] 

Although both isoenzymes catalyze the same reaction, heme oxygenase 1 and heme oxigenase 2 have distinctive functions in protecting tissue from damage.  Heme oxygenase 1 is rapidly induced by free radicals and hypoxia. The result is a rapid inactivation of pro–oxidant hem of denatured hemoproteins and conversion in bilirubin and carbon monoxide. In severe spinal cord injury, heme oxygenase 1 is induced and colocalizes with cyclic guanosine monophosphate (cGMP) and proapoptotic oncogenes. 

Besides the distinctive functions of the two isoenzymes, there is a different propensity for induction depending on the injury site. Induction of heme oxygenase 1 is found distal to the injury site, while glucocorticoid–inducible heme oxygenase 2 is present in the proximity of the cord lesion. Extremely high levels of messenger ribonucleic acid (mRNA) for heme oxygenase 1, and isoenzyme markers like Fe^3+^ and cGMP are found distal to the injury site, in about 4–16 hours after trauma. They colocalize within motor neurons below injury site with transcription factors and oncogenes: Fas–associated protein containing death domain (FADD), tumor necrosis factor–related apoptosis–inducing ligand (TRAIL) and p53. The result is the induction of FADD, TRAIL and p53 immunoreactivity distal to the injury site. On the other hand, the level of mRNA for heme oxygenase 2 was elevated in segments proximal to the injury site. Immunohistochemical analyses distal and proximal to the injury site show the presence of heme oxygenase 1 in distal segments. Heme oxygenase 1 positive neurons are found in the ventral horn in distal segments.[20] 

Another isoenzyme is heme oxygenase 3, whose transcription was recently reported in the central nervous system, and whose role in the spinal cord injury is unclear.[21]

Heme oxygenase 1 protects the tissue from progression of the lesion by promoting apoptosis, while heme oxygenase 2 is involved in the suppression of the inflammatory response induced by nitrosyl-derived radicals.[13,20] As a therapeutic strategy, studies done so far, suggest that the use of heme oxygenase in the treatment of spinal cord injury may be beneficial for the limitation of early vascular disturbances and in diminishing the inflammatory response.[22] 

## Inflammatory response

The spinal cord injury is followed by an inflammatory response, which pays an important role in the secondary pathogenesis and contributes to the progression of the secondary destructive phenomena, and also to the repairing processes.[23,24] 

The histopathological exam performed during the acute phase shows three distinctive areas: zone 1, including areas with inflammatory features, necrosis, and cystic cavities, zone 2, areas with axonal swelling, inflammation, and wallerian degeneration, and zone 3 histological intact. Zone 1 tends to increase in size with time after the injury, whereas the overall lesion, meaning zones 1 and 2 together, remain relatively constant in size, from the moment the lesion becomes macroscopically visible, usually in 1–3 days. Inflammatory cells are frequently encountered in zone 1, and sometimes in zone 2.[23]

### Inflammatory cells 

Spinal cord injury causes a primary injury and generates a cascade secondary pathogenic events, which lead to the progression of the tissue damage. [7,25–28] The cells involved in immune cellular response are: microglia, leucocytes (lymphocytes, neutrophils, monocytes), macrophages, and astrocytes. [7,28–27,29] The posttraumatic inflammation is characterized by the accumulation of microglia and macrophages, which contribute to the secondary pathogenesis.[7,26–28,30,31] Leucocytes and glial cells, mimic macrophages, causing tissue damage, myelin vesicles, and lipid peroxidation, through generating toxic molecules (free oxygen– and nitrosyl–derived radicals, cytokines and chemokines).[7,32] 

Posttraumatic hemorrhage leads to blood extravasation and infiltration of the spine with neutrophils. The early appearance of neutrophils exposed by the presence of human alpha neutrophilic defensin, at the contused cord site, represents a hemorrhage marker. The early inflammatory response is induced by neutrophilic signaling.[7,25,33] Neutrophils infiltrate the spine within the first hours after injury, have a peak at 24 hours [7,25,29,33,34], decrease in 48 hours [25], are rare in 7 days, but they can still be detected up to 10 days. [7,23,33] The increasing number of leucocytes is maximal during the first week after injury, and rarely persists after a week. [24] In 3 weeks time after the trauma the leucocytes disappear. [24] The neutrophils are preponderantly encountered in necrotic areas.[29] Neutrophils, measured by myeloperoxidase activity, are frequent to the injury site and in 4 mm length rostral and caudal from cord lesion. [29]  During the first 7 days after the injury, an increased number of leucocytes in the cerebrospinal fluid is seen, as a marker of the early immune response. 

A massive infiltration with B–lymphocytes and rare T lymphocyte is seen in 3–6 hours after the trauma. Lymphocytes B and T persist for 7 days.[7] 

Within the first days after the injury, an important number of microglia CD_68+_ and rare monocytes and macrophage are found. Activated microglia, monocytes, and macrophages persist weeks–months after the injury. Activated macrophages appear immediately after the trauma, but they have a peak in 2–4 weeks. [7,28] There is a rapid transformation of microglia in macrophages. [7,28]  Macrophages and microglia are exposed by anti–ED1 and OX–42 antibodies. They are encountered at 24 hours and have a peak at 48 hours. They are found predominantly in the gray matter and in the white matter in dorsal columns. Their number diminishes with increasing distance from the cord injury site. The number of macrophages and microglia is directly proportional with the severity of cord injury. [29] 

The cell capacity of causing oxidative or proteolytic damage is evidenced by the expression of the inflammatory cells of oxidative enzymes: myeloperoxidase, nicotin amid–adenine dinucleotidphosphat oxidase and matrix–metalloproteinase–9. The oxidative activity, measured by the activity of myeloperoxidase and nicotin amid–adenine dinucleotidphosphat oxidase is initially attributed to neutrophils and activated microglia. Usually, macrophages, do not express myeloperoxidase or nicotin amid–adenine dinucleotidphosphat oxidase. Matrix–metalloproteinase–9 is exclusively expressed by neutrophils. Therefore, destructive activity is maximal within the first days, and it exists due to neutrophils and activated macrophages. The anti–inflammatory treatment prevents neutrophils and macrophages inflow, the activation of microglia, phagocytic and secretory activity of macrophages, and can be administrated as a neuroprotective measure. [7,23,29] 

The inflammatory response causes axonal demyelination and neuronal death [7,31,32,35], but it can be involved in neural regeneration.[7,31,33,35] This hypothesis is supported by Carlson et al., who proved the correlation between the axial extend of the tissue damage and the number of macrophages and microglia.29 Macrophages and microglia promote axonal regeneration by scavenging myelin and neuronal debris[7,31,33,35], by producing proregenerative cytokines, as well as transforming growth factor beta (TGF-beta) [7,33], and by stimulating the neural growth.[7]

### Inflammatory mediators

Posttraumatic inflammatory response pays an important role in pathophysiological events. Among the inflammatory mediators, several pro–inflammatory and anti–inflammatory cytokines are found. Pro–inflammatory cytokines are represented by interleukin 1 beta, interleukin 6, tumor necrosis factor alpha (TNF–alpha), interleukin 2, soluble receptor for interleukin 2, and intercellular adhesion molecule 1 (ICAM–1).[36] Anti–inflammatory cytokines are receptor antagonist for interleukin 1 (IL–1RA), autoantibodies against myelin associated glycoprotein and anti–GM 1 (monosialogangliosid 1). 

The increased immunoreactivity of interleukin 1 beta, interleukin 6 and tumor necrosis factor alpha was detected in the neurons 30 minutes after the injury and, in neurons and microglia, 5 hours after the trauma, but the expression of these pro–inflammatory cytokines is short, and rapidly decreases to the basal level within 2 days after the injury. Activated microglia and axonal swelling can be detected during the early inflammatory response, in 30 minutes time after the injury. Numerous neutrophils are presented intramedullary, in the first days following the cord injury, and then their number deceases dramatically, while the macrophages have a progressive increase after the first day. So, intramedullary endogen cells, neurons and microglia, and not the blood leucocytes, are responsible from the early production of interleukin 1 beta, interleukin 6, and tumor necrosis factor alpha.[37] The increased cytokine level is not directly proportional with the number of leukocytes, cord injury level or ASIA (American Spinal Injury Association) classification.[38] 

The tumor necrosis factor is involved in the inflammatory response, as well as in the posttraumatic regeneration process.[39] An up–regulation effect in tumor necrosis factor alpha transport through blood–spinal cord barrier in spine cord injury was noted.[40] 

## Blood–spinal cord barrier breakdown

Posttraumatic blood–spinal cord barrier breakdown is an important cause of cord injury progression

### Blood–spinal cord barrier

Virchow–Robin space, subarachnoidian periarteriolar space, contains besides cerebrospinal fluid, pial and arachnoid cells with phagocytic behavior. The arteriole wall is composed of one layer of endothelial cells, surrounded by a smooth muscle coat. Capillaries lack the smooth muscle coat, their wall is composed of basal lamina, endothelial cell united through tight junctions and pericytes, muscular specialized cells. Astrocytic foot processes are in close contact with basal lamina. The blood-spinal cord barrier, located at the capillary level, prevents free passage of cells and different substances. Blood–spinal cord barrier has a double function: physical barrier and selective transportor.[41] The blood–spinal cord barrier controls the blood-spinal changes and achieves a stable environment, necessary to normal neural functioning.[41] Tight junctions prevent the passage of large molecules, like plasma proteins, and molecules carrying the same charge, like plasma proteins or glycoprotein–rich glycocalyx complexes, substances with anionic charge. [7,42] Besides, the blood–spinal cord barrier presents the selectivity for the transportation of different substances, keeping neural homeostasis. Blood–spinal cord barrier does not allow the passage of inflammatory cells.  

### Blood–spinal cord barrier breakdown 

Posttraumatic blood–spinal cord breakdown is biphasic. The primary mechanism is mechanic, damage secondary to forces generated by the traumatism, and has consequences like hemorrhage and ischemia. Later, different secondary pathophysiogical mechanisms, cytotoxicity of the inflammatory cells, and release of excitatory amino acids turned up. The secondary alteration of permeability of the blood-spinal cord barrier begins 3 days after the trauma and keeps up to 28 days.[8,39,40,43]  In this phase, the cord injury continues, breakdown of the barrier allowing inflammatory cells and toxic molecules inflow into the cord. Subsequently, the progression of the lesion, adjacent to initial contusion, in perihemorrhagic penumbra zone, appears. It is present not only to the injury site, but it extends along the spinal cord axis.[8] 

The blood–spinal cord barrier breakdown causes transitory loss of anionic–charge sites, having as a consequence the plasma proteins leakage. [8]The extravasation following minor and moderate trauma is minimal and usually limited to the injury site. In severe trauma, disruption of the barrier is more obvious centrally and into the gray matter. The axial extension of the barrier breakdown depends on the severity of the injury. Following the severe trauma, there is alteration of permeability in the dorsal columns up to 2 cm from the contusion level. [8]  The functionality of the blood-spinal cord barrier is reestablished in 3 hours–14 days after the moderate trauma. There is a peak of protein leakage in 3 hours–1 day, and the localization coincides with the hemorrhagic areas. In spite of hemorrhagic resolution, after day 7, the increased permeability for proteins, is maintained to the injury level, as well as in distant sites. Functional reestablishment is encountered after 14 days. [8]  Increasing posttraumatic protein levels lead to differential diagnosis problems with infection.[24] 

The disruption of blood–spinal cord barrier permits the exposure of the cord to the destructive effects of inflammatory cells.[41] When present in high quantities, the neurotransmitters such as glutamate and glycine, are cytotoxic. [41]  Post hemorrhage, a massive infiltration with leucocytes which cause injuries to neural structures, appears. 

Blood–spinal cord barrier breakdown has other negative effects. For example, the barrier breakdown causes a transient rise in blood pressure with irreversible consequences.[7,44,45] Blood–spinal cord barrier disruption induces stress proteins such as heat shock proteins 32 and 70 (HSP 32 and HSP 70), markers of tissue injury and oxidative stress.[13,17,46] 

### Factors implicated in increasing permeability of blood–spinal cord barrier

Secondary pathophysiological mechanisms, which contribute to the disruption of blood–spinal cord barrier and the progression of the traumatic lesions, are represented by the exposure to a large spectrum of substances such as cytokines, vasoactive peptide (endothelin 1).

#### Endothelin 1

3 isoforms have been identified: endothelin 2, endothelin 3 and vasoactive intestinal constrictor polypeptide (VIC). 

Endothelin 1 mediates the oxidative stress by modulating blood flow to the spine.[18] There are 3 receptors for endothelin: ET_A_, ET_B1_ and ET_B2_.[7,47] The ET_A_ receptor is localized in the smooth muscle coat and produces vasoconstriction. The ET_B_ receptor has two subtypes of receptors: ET_B1_ and ET_B2_. ET_B1_ is localized in the endothelial cells and induces vasodilatation, and ET_B2_ is present in the smooth muscle coat and produces vasoconstriction. Endothelial actions on injured cord are executed through ET_A_ and ET_B2_ receptors. ETA produces vasospasm, secondary to subarachnoid hemorrhage, prolonged vasoconstriction, and ischemic damage.[47] There are three probable sources of endothelin 1 following hemorrhage. The first source is represented by the humoral compartment. High after the subarachnoid hemorrhage, Endothelin 1 gets into the central nervous system through the disrupted barrier. The second source is myelomalacia, and the third source are the eritrocits.[7] Endothelin 1 produces prolonged vasospasm, ischemic damage and blood–spinal cord barrier breakdown.[7,48] Increasing endothelin expression at contused cord, correlates with the blood–spinal cord barrier breakdown.[49] Intrathecal administration of endothelin generates free radicals.[50]  Intrathecal administration of endothelin 1 in normal cord, results in barrier breakdown.[48] Endothelin 1 induces heme oxygenase 1 in dorsal columns astrocytes.[18] 

The therapeutic strategies, using endothelin 1 blocking agents, that impede endothelin 1–mediated vasoconstriction, are beneficial in stopping cord injury progression.

#### Metalloproteinase

Matrix–metalloproteinases are zinc– and calcium–depended endopeptidases, which mediate extracellular degradation of the matrix, by hydrolyzing the matrix components.  The matrix–metalloproteinase release growth factors and cytokines from the matrix. Experimental studies showed that matrix–metalloproteinases are involved in the inflammatory response, increasing permeability of the blood–spinal cord barrier, and ischemia–induced angiogenesis. 

During early inflammatory response neutrophils, monocytes and macrophages infiltrate the injured cord segment. These cells express matrix–metalloproteinase such as matrix–metalloproteinase–2 (gelatinase A), matrix–metalloproteinase–8 (neutrophil collagenase), matrix–metalloproteinase–9 (gelatinase B), matrix–metalloproteinase–11 (stromelysin–3), and matrix–metalloproteinaza–12 (metalloelastase). They participate in infiltration, migration, tissue damage, matrix degradation, blood–spinal barrier breakdown and edema.[51] Matrix–metallopreteinases degrade basal lamina, leading to increased permeability of the blood–spinal cord barrier. During the inflammatory response and angiogenesis, the basal lamina is deteriorated and the barrier is permeable. The injection of pro–inflammatory cytokines or tumor necrosis factor alpha induces the matrix–metalloproteinase–9 (gelatinase B) expression. Matrix–metalloproteinase blocking agents may be useful in the early treatment of spinal cord injury. 

Angiogenesis is a response to hypoxia. Matrix–metalloproteinase–1 is found on endothelial cells and is required for angiogenesis. Hypoxia and ischemia are results of direct damage of the blood vessels or of the posttraumatic hypoperfusion and vasodilatation. Proangiogenic factors released from the injury site rapidly increase vascular permeability. Fibrinogen is polymerized into fibrin. Endothelial cells require matrix–metalloproteinase–1 for matrix degradation and chemotactism. The increased levels of matrix–metalloproteinase–1, matrix–metalloproteinase–2 and matrix–metalloproteinase–9 are found in angiogenesis. Increased vascular endothelial growth factor (VEGF) expression, proangiogenic factor, correlates with matrix–metalloproteinase–2 and matrix–metalloproteinase–9 expression, and has an up–regulation effect for matrix–metalloproteinase–9.[7,51] 

Matrix–metalloproteinase proteolysis has the following consequences: invasion of the endothelial cells into the surrounding areas, by the degradation of the extracellular matrix, which has chemotactic effects for endothelial cells, activation and release of the growth factors, and increased permeability of the blood–spinal cord barrier. 

The role of angiogenesis in spinal cord injury is still on debate. Nevertheless, there is data which suggest that the inhibition of angiogenesis might have neuroprotective effects. 
